# Circadian acclimatization of performance, sleep, and 6-sulfatoxymelatonin using multiple phase shifting stimuli

**DOI:** 10.3389/fendo.2022.964681

**Published:** 2022-11-16

**Authors:** Shawn D. Youngstedt, Jeffrey Elliott, Salma Patel, Natalie Zi-Ching Mak, Evan Raiewski, Elias Malek, Michael Strong, Chung Jung Mun, Tyler Peters, Remun Madlol, Natasha Tasevska, Massiullah Rasoul, Cindy Nguyen, Kimberly M. Vargas Negrete, Andejola-Omobonike Adaralegbe, Sruthi Sudalaimuthu, Delaney Granholm, Anastasia Finch, Aryan Eksambe, Anannya Malready, Sairam Parthasarathy

**Affiliations:** ^1^ Edson College of Nursing and Health Innovation, Arizona State University, Phoenix, AZ, United States; ^2^ Department of Medicine, University of Arizona, Tucson, AZ, United States; ^3^ Department of Psychiatry, University of California, San Diego, La Jolla, CA, United States; ^4^ Department of Psychology, California State University, San Marcos, CA, United States; ^5^ Department of Kinesiology and Nutrition Sciences, Univeristy of Nevada, Las Vegas, NV, United States; ^6^ Department of Psychiatry and Behavioral Sciences, Johns Hopkins University, Baltimore, MD, United States

**Keywords:** jet lag disorder, bright light, exercise, melatonin, circadian misalignment

## Abstract

Misalignment between the environment and one’s circadian system is a common phenomenon (e.g., jet lag) which can have myriad negative effects on physical and mental health, mental and physiological performance, and sleep. Absent any intervention, the circadian system adjusts only 0.5-1.0 h per day to a shifted light-dark and sleep-wake schedule. Bright light facilitates circadian adjustment, but in field studies, bright light is only modestly better than no stimulus. Evidence indicates that exercise and melatonin can be combined with bright light to elicit larger shifts but no study has combined all of these stimuli or administered them at the times that are known to elicit the largest effects on the circadian system. The aims of this study are to compare the effects of different treatments on circadian adjustment to simulated jet lag in a laboratory. Following 2 weeks of home recording, 36 adults will spend 6.5 consecutive days in the laboratory. Following an 8 h period of baseline sleep recording on the participant’s usual sleep schedule on Night 1 (e.g., 0000-0800 h), participants will undergo a 26 h circadian assessment protocol involving 2 h wake intervals in dim light and 1 h of sleep in darkness, repeated throughout the 26 h. During this protocol, all urine voidings will be collected; mood, sleepiness, psychomotor vigilance, and pain sensitivity will be assessed every 3 h, forehead temperature will be assessed every 90 min, and anaerobic performance (Wingate test) will be tested every 6 h. Following, the circadian assessment protocol, the participant’s sleep-wake and light dark schedule will be delayed by 8 h compared with baseline (e.g., 0800-1400 h), analogous to travelling 8 times zones westward. This shifted schedule will be maintained for 3 days. During the 3 days on the delayed schedule, participants will be randomized to one of 3 treatments: (1) Dim Red Light + Placebo Capsules, (2) Bright Light Alone, (3) Bright Light + Exercise + Melatonin. During the final 26 h, all conditions and measures of the baseline circadian protocol will be repeated. Acclimatization will be defined by shifts in circadian rhythms of aMT6s, psychomotor vigilance, Wingate Anaerobic performance, mood, and sleepiness, and less impairments in these measures during the shifted schedule compared with baseline. We posit that Bright Light Alone and Bright Light + Exercise + Melatonin will elicit greater shifts in circadian rhythms and less impairments in sleep, mood, performance, and sleepiness compared with Dim Red Light + Placebo Capsules. We also posit that Bright Light + Exercise + Melatonin will elicit greater shifts and less impairments than Bright Light Alone.

## Introduction

### Circadian rhythms

**Figure 1:**
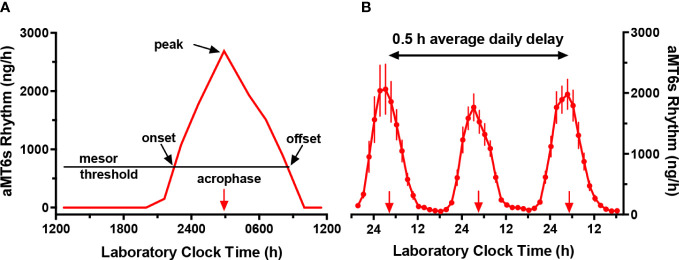
Circadian rhythms of urinary 6-sulfatoxymelatonin excretion (aMT6s, ng/h) in laboratory 2 h wake: 1 h sleep schedules repeated for > 24 h. **(A)** Schematic figure illustrating an individual baseline rhythm showing times of aMT6s onset (evening rise) and offset (morning decline), each measured, respectively, from upward and downward crossings of the cosine mesor (threshold). Also indicated are the cosine acrophase (time of mean vector of the cosine fit) and the time of the nighttime peak (the single highest ng/h value in the 24 h profile). **(B)** Robust aMT6s rhythm observed in young subjects studied in a 2 h wake: 1 h sleep schedule for 72 h. Under these conditions, in the absence of 24 h cycles in key environmental time cues, successive times of aMT6s onset and acrophase delayed on average 0.5 h per day from nights 1 to 3 (plotted points represent means ± 95% CI, n=17), demonstrating regulation by an internal endogenous circadian clock demonstrating regulation by an internal endogenous circadian clock. Normally, aMT6s and other circadian rhythms are synchronized to a period of 24 h by the 24 h cycles of our environment.

Circadian rhythms are near-24 h oscillations in physiology and behavior (1), which are controlled by an internal pacemaker located in the suprachiasmatic nucleus in the brain. Over the past 30 years, it has been recognized that melatonin and its urinary metabolite [6-sulfatoxymelatonin (aMT6s)] are the most robust measures of the central human circadian pacemaker (2, 3). **Figure 1** displays features of the aMT6s rhythm which are commonly measured (A), and the expected gradual daily delay in phase (B) when participants are separated from environmental and behavioral time cues that synchronize the circadian system to 24 h.

**Figure 2:**
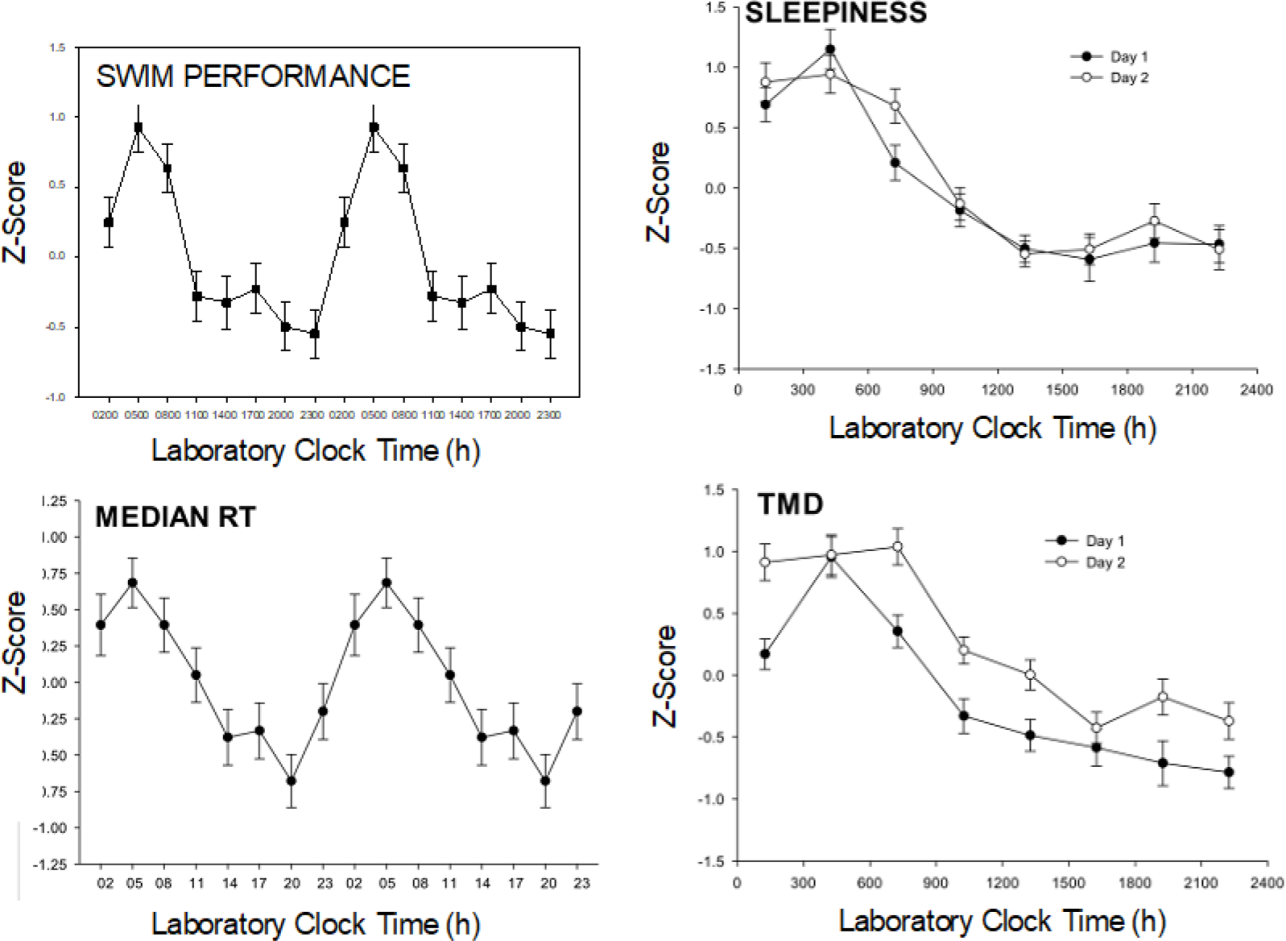
Example circadian rhythms. Data are from a study by Kline et al. N = 25 young adults followed an ultrashort sleep wake cycle (2 h wake: 1 h sleep) for 55 hours. Six 200 meter swim trials were performed at the indicated lab clock times throughout the day and night portions of the subject’s internal circadian rhythms (8 times/24 h). Similarly, the PVT (median reaction time RT), Profile of Mood States (Total Mood Disturbance: TMD) and Stanford Sleepiness Scale were measured every wake period and the data were z-transformed (normalized). The swim performance and reaction time data are “double-plotted” after averaging over the 2 days to better show circadian patterns. Strong circadian rhythms were found in each of these variables with similar patterns observed on Day 1 and Day 2 (right two panels).

Demonstration of circadian rhythms requires chronobiological techniques that separate rhythm measurements from environmental and behavioral factors which might “mask” their measurement, such as light and dark, sleep and wake, food intake, physical activity, and posture. These “unmasking” techniques have included the constant routine involving continuous bedrest (without sleep) and bedpans for 24-30 hours (h) (4), forced desynchrony protocols involving 3-4 weeks of recording on, for example a 30 h day (20 h wake-10 h sleep) (5), and the ultrashort sleep wake schedule involving, for example, 2 h of wakefulness and 1 h sleep repeated over ≥ 24 h (6). The ultrashort sleep-wake schedule distributes masking factors equally across the 24 h day. The ultrashort sleep-wake schedule has been the technique of choice for several of our studies (and the present study) (7–10), partly because it is relatively noninvasive and involves little sleep loss (compared with the constant routine), and because it is relatively short in duration (compared with forced desynchrony protocols). **Figure 2** displays circadian rhythms of swim performance, sleepiness, psychomotor vigilance, and total mood disturbance established using the ultrashort sleep-wake schedule (7, 8).

### Circadian misalignment in civilians and military

Under usual conditions, the circadian system is synchronized to promote optimal functioning in the environment (11). This synchronization is mediated by the 24 h periodicity of the environments. Exposure to the diurnal light/dark schedule is thought to be the primary synchronizing stimulus. In humans, circadian synchronization is evidenced by optimal mental and physiological functioning in the daytime and sound sleep at night. However, misalignment between the environment and one’s circadian system is a common phenomenon with myriad negative consequences.

For example, approximately 20% of the world’s work force are shift-workers who have a higher prevalence of cardiovascular disease (12), cancer (13), obesity (14), depression (15), disturbed sleep, and accidents and errors (16) (e.g., a nurse giving the wrong medicine or wrong dose of medicine to a patient) compared with regular day workers. Chronic exposure to rapid transmeridian travel has been associated with cognitive deficits (17) and mood disturbance in humans (18), and with reduced longevity in animal models (19). Moreover, “social jet lag”, characterized by delayed sleep timing on free days relative to work days (20), has been associated with obesity (20), cardiometabolic risk, and impaired glucose regulation.

Circadian misalignment is highly prevalent among military personnel, due to rapid transmeridian deployment, night operations, and shiftwork, which often occur simultaneously (21). Special Forces must be prepared to transport anywhere in the world within 18 h of executive notification, and night operations are embraced by the US military due to their technological superiority and goals of stealth. These forces must be ready to function at the highest physical and mental level with dire risks associated with error or slowness in reaction time or decision-making. Without effective countermeasures, these missions are likely to entail decrements in performance related to circadian misalignment. Additionally, chronic exposure to circadian misalignment could contribute to PTSD (22), depression (22), diabetes (23), obesity (14), and other comorbidities among veterans.

### Current approaches for circadian acclimatization are inadequate

Absent any specific intervention, the circadian system adjusts only about 0.5-1.0 h per day to a shifted light-dark and sleep-wake schedule. Moreover, even full-time night workers almost never fully adjust to night work because of exposure to stimuli which keep their body clocks on a diurnal schedule during the work week, and because night workers typically revert back to diurnal behavior on their free days (24). Until circadian acclimatization occurs, individuals experience disturbed sleep, impairments in performance, alertness, and mood, and other symptoms such as gastrointestinal distress.

In the military, some success has been realized, for example, in improving the notoriously problematic sleep and work schedules of crew members aboard Navy ships (25), and using caffeine and other stimulants to promote mental and physiological performance during the night and during operations involving periods of prolonged wakefulness (26, 27). However, these approaches have mostly targeted the symptoms of circadian misalignment; they have had little impact on circadian acclimatization, the resynchronization of the circadian system. Moreover, stimulants lose much of their efficacy after a few days of use (28), and chronic stimulant use has been associated with a higher risk of fatigue, stress, and burnout (29). As in the civilian world, what is urgently needed are techniques for eliciting dramatic, essentially complete, circadian acclimatization, preferably techniques which can be applied safely to alleviate chronic circadian misalignment.

**Figure 3:**
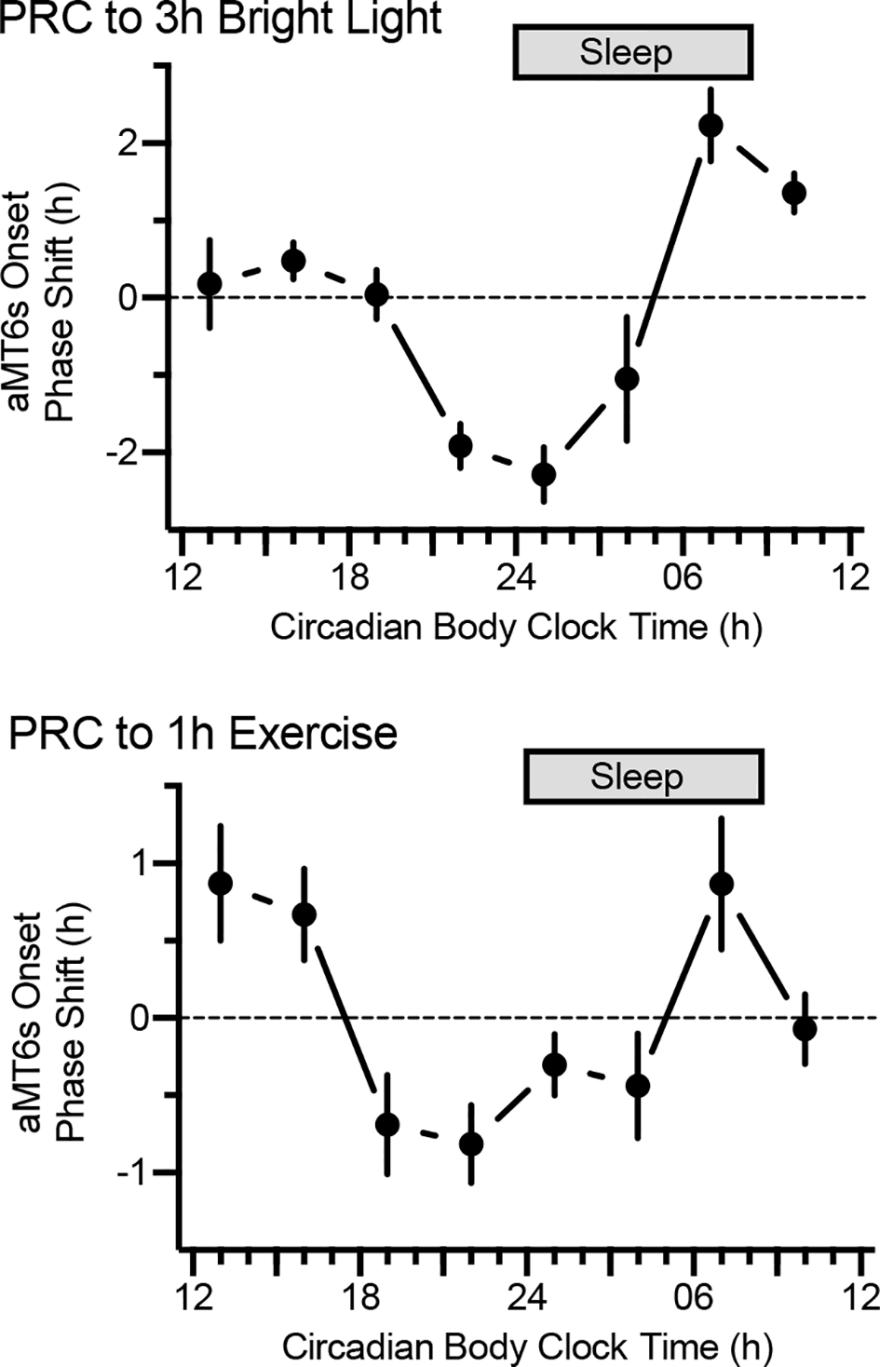
Phase-response curves to bright light (n = 105, top) and exercise (n = 101, bottom). By convention, phase advances and phase delays are denoted by positive and negative values on the ordinate, respectively. Participants followed the ultrashort sleep-wake schedule for 5.5 days, receiving light (3 h, 5,000 lux) or exercise (1 h, moderate) on 3 consecutive days. Shifts in aMT6 onset from baseline to end-of-study (y axes) depended on timing of the stimuli. Note: maximal phase delays for light and exercise occurred at 0100 h and 2200 h circadian body clock time, respectively.

The most fundamental chronobiological tool for informed correction of circadian misalignment is the phase-response curve which describes the direction and magnitude of shifts in the circadian system depending upon the timing of exposure to phase-shifting stimuli (zeitgebers) (11). For example, as shown by our research team (**Figure 3**) and others, the phase-response curve for bright light is characterized by phase delays (shift to a later time, helpful for westward travel) to bright light in the late night/early morning, phase advances (shift to an earlier time, helpful for eastward travel) to morning bright light, and small effects of light exposure during the afternoon (9, 30).

Bright light is regarded as the most important zeitgeber. However, although bright light can elicit large shifts under extreme laboratory conditions, such as with continuous bedrest and daily exposure to 5 h of 10,000 lux light (31), two comprehensive reviews have concluded that in field conditions bright light is only minimally effective compared with no treatment for shifting the circadian system or for ameliorating the symptoms of jet lag (32, 33). Thus, over the last quarter century there have been limited advancements in research or application directed at achieving more effective circadian acclimatization. We attribute this shortcoming to a nearly exclusive research focus on artificial bright light.

Far greater circadian acclimatization could be realized by combining bright light exposure with other known zeitgebers. Clearly, the three best-established zeitgebers in humans are bright light, exercise, and exogenous melatonin (34, 35). Research by our group (10, 36–38) and others (39–43) indicates that exercise elicits significant circadian phase shifts. Our team has demonstrated significant effects with 1 to 1.5 hours of moderately intense exercise, and we recently demonstrated a significant phase-response curve (PRC) for exercise (**Figure 3**) (10).

A clear PRC for melatonin has been established with peak phase-advancing and phase- delaying effects at 1800 h and 0500 h, respectively, nearly the inverse of the timing of optimal shifts by bright light (34). The optimal melatonin dose for shifting the circadian system is 0.5mg (44); apparently higher doses result in melatonin staying in the bloodstream long enough to also have an effect on the opposing portion of the melatonin PRC, thereby reducing the effectiveness of the exogenous melatonin to shift the circadian clock in the desired direction.

Research has shown additive effects of bright light combined with melatonin (45, 46) or exercise (37, 38). However, no study has combined all three zeitgebers, nor have studies combined these stimuli at the optimal times based on their respective phase-response curves.

Special Forces and other individuals have often attempted intuitive solutions such as exercising immediately upon arrival at an overseas destination. Depending on the time of day and time zones crossed, such practices can indeed promote circadian acclimatization, but in other scenarios, these practices are likely to have no effect or could hinder circadian acclimatization. This problem can now be readily remedied by applying the precise stimulus timing described in our separate publications of complete phase response curves for bright light and exercise (**Figure 3**).

Demonstration of full and rapid restoration of circadian alignment would have a significant impact on research and application. For example, if within just a few days of travel to the other side of the planet military forces could be prepared for physical and mental challenges, then the advantages over current approaches which focus on shifting the sleep-wake cycle or light exposure, would be considerable. Likewise, the ability to alternate between day and night operations with minimal impairment would provide a significant advance for warfighters. Civilians could have analogous benefits.

The aims of this study are to compare the effects of three different experimental treatments: (1) Dim Red Light + Placebo Capsule, (2) Bright Light, and (3) Bright Light + Exercise + Melatonin on circadian acclimatization to an 8 h delay of the sleep/wake and light/dark over 3 days. Circadian acclimatization will be defined by phase delays in various circadian rhythms and by reduced impairment in sleep and functioning (each described below).

## Methods

The study was reviewed and approved by the Institutional Review Board (Human Subjects Protection Program) at the University of Arizona and the Human Research Protection Official of the Department of Defense (United States Special Operations Command). The participants provided their written informed consent to participate in this study.

### Design overview

**Figure 4:**
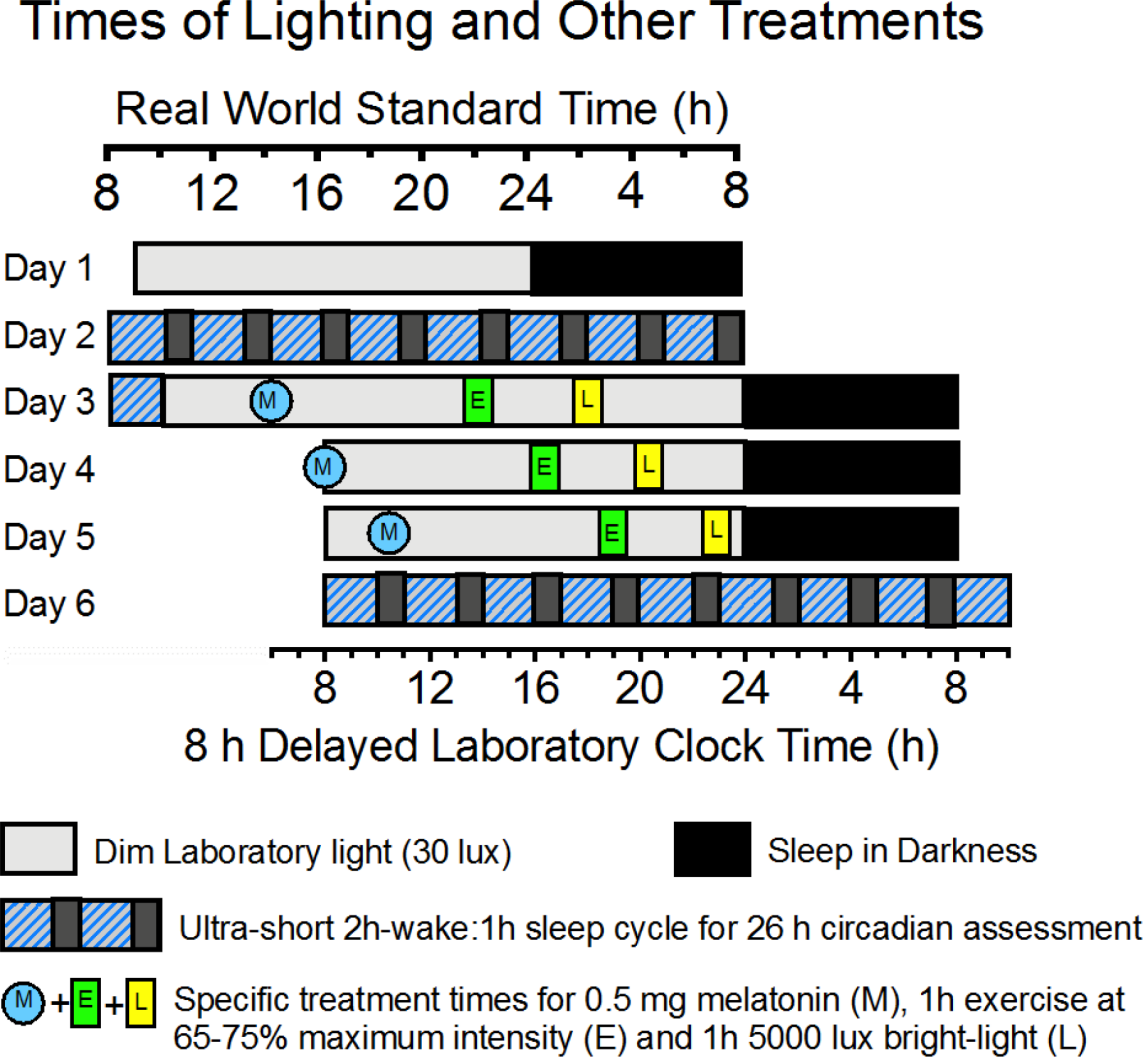
Schematic of laboratory light:dark cycles and timing of scheduled melatonin (M), exercise (E), and bright Light (L) treatments specified in the text and **Table 1**. The figure depicts specifics only for the Melatonin + Exercise + Bright-Light treatment For consistency, all times in **Table 1** are real world Arizona Standard Time, even after the 8 h delay of Laboratory Clock Time (bottom x-axis).

Following a 2 week home baseline assessment, 36 individuals will spend 6.5 days in the laboratory (**Figure 4** and **Table 1**). Following an 8 h baseline sleep assessment on Night 1 of the 6.5 day lab period, participants will undergo a 26 h baseline circadian assessment *via* an ultrashort sleep-wake schedule involving 2 h wake intervals and 1 h sleep intervals, repeated throughout the 26 h assessment. Following baseline circadian assessment, for the next three 24 h days, participants will adhere to a 16 h wake-8 h sleep schedule in which the wake-sleep and light-dark schedule are delayed 8 h relative to their home schedules (analogous to traveling 8 time zones west). Participants will be randomized to one of 3 treatments (n=12 per treatment) which will be administered on each of the 3 days of the shifted (8 h delayed) schedule: (1) Dim Red Light + Placebo Capsules, (2) Bright Light Alone, and (3) Bright Light + Exercise + Melatonin. Following the last 8 h sleep period of the shifted schedule, participants will be assessed with an end-of-study 26 h ultrashort sleep wake schedule. On baseline Day 1 and Days 1-3 of the shifted schedule, sleepiness, mood, mental performance, and pain sensitivity will be assessed every 3 h during wake. During all 8 h sleep periods, sleep will also be recorded with the Z-machine, which assesses sleep stages from 3 EEG electrodes. During both 26 h ultrashort sleep-wake schedules, mental performance, physiological performance, urinary aMT6s, mood, sleepiness, and pain sensitivity be measured around-the-clock.

**Table 1:** Experimental protocol.

Day 1	Arrive in lab at 0900 PVT assessed at 1115, 1415, 1715, 2015, and 2315 Sleepiness and mood assessed at 1120, 1420, 1720, 2020, and 2320 Pain threshold test at 1130, 1430, 1730, 2030, 2330
Day 2	Z-machine recorded sleep at 0000-0800 Ultra-short sleep-wake cycle 0800-2400 Collection of every urine voiding for aMT6s6 Forehead temperature assessed at 0810, 0950, 1110, 1250, 1410, 1550, 1710, 1850, 2010, 2150, 2310 PVT assessed at 0815, 1115, 1415, 1715, 2015, 2315 Mood and sleepiness assessed at 0820, 1120, 1420, 1720, 2020, 2320 Pain senstitivy at 0830, 1130, 1430, 1730, 2030, 2330 Wingate Test at 0900, 1500, and 2100
Day 3	Ultra-short sleep wake cycle from 0000-1000 Every urine voiding collected for aMT6s from 0000-1000 Forehead temperature at 0050, 0210, 0350, 0510. 0650, 0810, 0950 PVT assessed at 0215, 0515, 0815, 1115, 1415, 1715, 2015, 2315 Mood and sleepiness assessed at 0220, 0520, 0820, 1120, 1420, 1720, 2020, 2320 Pain sensitivity at 0230, 0530, 0830 Wingate Trialsat 0300 Melatonin capsule at 1400 Exercise at 2130-2230
Day 4	Bright Light 0130-0230 PVT assessed at 0215, 0515, 1715, 2015, 2315 Sleepiness and mood assessed at 0220, 0520, 1720, 2020, 2320 Pain sensitivity at 1730, 2030, 2330 Z-machine recorded sleep 0800-1600 Melatonin capsule at 1630
Day 5	Exercise at 0000-0100 PVT assessed at 0215, 0515, 1715, 2015, 2315 Bright Light 0400-0500 Sleepiness and mood assessed at 0220, 0520, 1720, 2020, 2320 Pain threshold at 0230, 0530, 1730, 2030, 2330 Z-machine recorded sleep at 0800-1600 Melatonin Capsule at 1900
Day 6	PVT assessed at 0215, 0515, 1715, 2015, 2315 Sleepiness and mood assessed at 0220, 0520, 1720, 2020, 2320 Exercise at 0230-0330 Pain sensitivity at 0530, 1730, 2030, 2330 Bright Light at 0630-0730 Z-machine recorded sleep 0800-1600 Ultrashort sleep wake cycle from 1600-2400 Every urine voiding collected from 1600-2400 for aMT6s Forehead tempeature 1610, 1750, 1910, 2050, 2210, 2350 Wingate test at either 1700 and 2300 or 2000, or 2300
Day 7	Ultra-short sleep wake cycle from 0000-1800 Every urine voliding collected for aMT6s Forehead tempeature at 0150, 0310, 0450, 0610, 0750, 0910, 1050, 1210, 1350, 1510, 1650 Wingate test at 0500 and 1100 PVT assessed at 0215, 0515, 0815, 1115, 1415, 1715 Mood and sleepiness assessed at 0220, 0520, 0820, 1120, 1420, 1720 Pain sensitivity at 0230, 0530, 0830

Times below are for baseline sleep of 0000-0800 h, and the Bright light + Exercise + Melatonin treatment on days 3-5. Four trials of the Wingate test will each be separated by 6 hours. The timing of the trials will be staggered with subjects performing the first trial at 0900, 1200, or 1500. Please note: All the times noted are the Arizona time, including after the 8 h delay of the sleep-wake and light dark schedul (starting on day 3). The example is provided for a subject with a baseline sleep schedule of 0000-0800 who was assigned to complete the first Wingat Trial at 0900 on Day 2.

### Why are the lab protocols 6.5 days?

In our view, 3 days is as quickly as could be expected to shift the circadian system by up to 8 h in response to an 8 h delay of a “normal” 16 h/8 h wake/sleep dim-light/dark cycle. A very careful examination of the circadian system and how it shifts to the stimuli requires at least 24 h before and 24 h after the treatment (plus a couple of extra hours to minimize “transient” carry-over effects of treatments on measurements of circadian rhythm phase-shifts or changes in waveform).

### Why a delayed schedule instead of an advanced schedule?

Circadian acclimatization to both delayed and advanced schedules is important to assess. The results of the present study will have particular implications for adapting to a nighttime work schedule as well as for westward travel.

### Recruitment

Thirty-six individuals (plus dropouts) ages 18-50 years will be recruited using social media, newspaper advertisements and flyers in the Tucson and Phoenix areas. Our goal is to recruit an equal number of women and men participants who are representative of the race/ethnicity of the population of Tucson and Phoenix.

### Inclusion and exclusion criteria

The participant inclusion criteria include the following: (1) ages 18-50 years, (2) currently participate in moderate levels of physical activity, (3) ability to speak and read English proficiently.

Moderate levels of physical activity are defined as performing aerobic exercise sessions that occur at least 2 days per week, for a minimum of 20 minutes, at an intensity of at least 60% of their maximal effort. Moderate levels of physical activity further establish the safety of the study and the ability to handle the treadmill and Wingate anaerobic performance aspects of the study (described below). The English speaking requirement is based upon limitation of the staff, and because the study surveys are written in English.

The participant exclusion criteria include the following: (1) having more than one major risk factor for coronary artery disease; (2) having any symptom or sign of cardiopulmonary disease; (3) recent shift-work experience (previous 2 months) or travel across multiple time zones (previous 4 weeks); (4) having an abnormal sleep-wake schedule (i.e., reported bedtime before 9:00 pm or after 2:00 am; wake time before 5:00 am or after 10:00 am); (5) being an extreme night owl or morning lark, as assessed by the Horne-Ostberg Morningness-Eveningness Scale; (6) having sleep apnea (as assessed with the STOP-BANG questionnaire) or another sleep disorder; (7) having depressed mood [Center for Epidemiologic Studies-Depression Scale (CES-D) > 16]; (8) the use of medications likely to distort melatonin excretion or cardiovascular responses to exercise; (9) the use of sleeping pills or melatonin more than once per week; (10) having high sensitivity to light; (11) the abuse of alcohol or drugs; and (12) having any physical or mental health condition that would contraindicate participation in exercise or the other rigors of the experiment.

### Screening procedures

Screening will involve a multi-step process of informing individuals about the study and ensuring safety and appropriateness of the study. Most prospective participants will scan a link on a study ad or flyer which will prompt them to complete an initial interest form which will then be stored in a secure database (REDCap, Vanderbilt University, Nashville, TN).

The next step will be a brief phone screen. This will further establish interest and eligibility.

Interested prospective participants who pass the phone screen will visit the study research office. After an extensive study orientation, they will be invited to sign informed consent and the they will complete several initial screening questionnaires, including the Physical Activity Readiness Questionnaire, the Center for Epidemiologic Studies-Depression scale, the Horne-Ostberg Morningness-Eveningness Questionnaire, the STOP-BANG sleep apnea questionnaire, and a medication questionnaire. Participants must be classified as low risk for participation in moderate-intensity exercise, following the American College of Sports Medicine and American Heart Association guidelines. Participants will then practice the psychomotor vigilance test (described below) and practice exposure to the pressure pain threshold test (described below).The next screening step will involve a submaximal treadmill test to estimate maximal oxygen uptake (VO2max) and ensure exercise capacity for the completing the study. Participants will also practice two trials of the Wingate Anaerobic Performance test (described below).

### Home baseline

During a 14 day period immediately prior to the 6.5 day lab protocol, participants will be asked to follow a stable sleep-wake schedule consisting of ≥ 7 h time-in-bed, ≥ 6 h of sleep per night, and bedtime and wake-time not varying by > 60 min per night on average. Adherence to this sleep schedule, and confirmation of normal sleep patterns will be verified by continuous wrist actigraphic recording, supplemented with a daily sleep diary, which will serve as a final screen for participation. Participants will also keep a daily physical activity log. Prior to this baseline, participants will complete a food preferences questionnaire that will be used to provide meals while they are in the laboratory portion of the study (described below).

### Laboratory assessment

Within 48 h of the home baseline, participants will arrive at the laboratory between 0800-11000 on laboratory Day 1, and they will stay in the laboratory for 6.5 days (**Figure 4**, and **Table 1**). Laboratory light levels will be kept at 30 lux during all wake periods (except during the bright light treatment) and <0.5 lux during all sleep periods. Blue light and dimming filters will be used on electronic devices that the participants use. Outside of designated sleep periods, sleep will be prevented with continuous monitoring of the participants. During the laboratory protocol, participants will be free to use their laptop computers and cell phones, read, visit with staff (outside of the sleep periods), etc. Participants will be free to ambulate around the laboratory, but exercise will not be permitted except as prescribed for the study. Participants will not be permitted to use alcohol or caffeine. Gradual tapering from these substances will be encouraged prior to entering the laboratory.

All meals during the laboratory stay will be provided to participants and will be consistent with each participant’s usual diet. Meals will be prepared by staff. Food quantities and energy intake will be based on the participants’ energy requirements determined using the Mufflin-St. Jeor equation (47), their physical activity level, and prescribed exercise during the laboratory study to ensure that participants remain in energy balance. Participants will be instructed not to eat or drink anything prepared outside the experimental kitchen, besides water and non-caloric drinks.

### Baseline Z-machine recorded sleep

On Night 1 in the laboratory, participants will have an 8 h period of sleep recording timed to coincide with their usual sleep schedule (described below).

### Baseline ultrashort sleep-wake schedule

Upon arising on Day 2, participants will follow an ultrashort sleep-wake schedule for 26 h (Days 2-3). The schedule will consist of alternating intervals of 2 h of wake in 30 lux light and 1 h of attempted sleep (monitored by Z machine) in <0.5 lux light, repeated throughout the 26 h (**Figure 4**). During each wake period (9 in total), participants will be provided with small meals/snacks equivalent to 1/8 of their daily caloric and macronutrient intake. For the present study, we consider that this schedule is preferable in terms of cost, relatively low participant burden, ability to provide the desired performance data, and minimal sleep loss (subjects still sleep 5-6 h per 24 h period) compared with other circadian rhythm measurement protocols. As shown in **Figures 1** **–** **3**, the ultrashort sleep-wake schedule has been used successfully to measure circadian rhythms in multiple studies (7–10). Careful measurement of shifts in the circadian system, especially in aMT6s, and in rhythms in behavioral outputs of mental performance, physiological performance, mood, and, and sleepiness (see below), will provide a good indication of circadian acclimatization.

### Shifted wake-sleep, light-dark, and meal schedule

On laboratory Day 3, the participants’ sleep-wake and light-dark schedules will be delayed by 8 h, analogous to flying westward across 8 time zones. Following the shift, participants will commence a 16 h wake-8 h sleep and light-dark schedule which is delayed 8 h in comparison with the participants’ home schedules. Thus, subjects will be awake for 8 h longer than usual on the first day of this shift. Timing of meals and snacks will also be delayed 8 h. This shifted schedule will be maintained for 3 days (Days 3-5).

### Experimental treatment randomization

Participants will be randomly assigned to 1 of 3 treatments (n=12 per treatment) to be administered on the three days of the delayed laboratory schedule. The randomization will be stratified based on the participants’ sex and whether they have high or low scores on the Horne-Ostberg Morningness-Eveningness Questionnaire (48, 49). Both participants and staff will learn of the randomization at the time that a participant enters the laboratory. Blinding of staff to treatment does not seem feasible since the staff will administer the treatments. Below are treatment times corresponding with the delayed schedule for a participant with a sleep schedule from 0000-0800 h. Following treatment assignment, expectancy for improvement in mood, sleep, and performance will be measured by 5-point Likert scales.

### Bright light alone

For this treatment, participant will wear Re-Timer light glasses for 1 h. Exposure will be verified by continuous observation by staff. During exposure, the participants may read, work on a laptop computer, etc. The Re-Timer glasses have been shown to shift the circadian system and reduce depression (49).

For a participant who sleeps from midnight to 8 am, the treatment will begin at 1730 h on Day 3 (midpoint at 1800 h), which corresponds with 0130 h on the baseline Arizona time schedule, a time of maximal phase-delaying effects of light (**Figure 3**). We expect that the bright light treatment combined with the schedule shift will elicit phase shifts of approximately 1.5 h per day. To accommodate this shift, the bright light exposure will begin at laboratory time 1900 (Arizona time: 0300) on Day 4 and 2030 on Day 5 (Arizona time: 0430), such that the light continues to occur at the maximal phase delay region of the phase response curve each day.

### Bright light + exercise + melatonin

The bright light in this treatment will duplicate that of the Bright Light Alone treatment. Participants will also perform 1 h of moderate-intensity treadmill exercise (55-70% of heart rate reserve), beginning at 1330 h, 1600 h, and 1830 h laboratory shifted times on Days 3-5, respectively (Arizona times 21:30 h, 0000 h, and 0230 h, respectively). Participants will consume 0.5 mg melatonin tablets (prepared by a local pharmacy) at laboratory times 0600 h, 0830 h, and 1100 h on Days 3-5, respectively. These times correspond to maximal phase-delaying effects of these three stimuli (**Figure 3**). We expect phase delays approaching 2.5 h per day in this treatment. As with Bright Light Alone, the timing of bright light, exercise, and melatonin will be delayed each day so that they occur at the maximal phase delay region of the respective PRCs for these stimuli. The proposed exercise and melatonin stimuli have elicited significant circadian phase delays (10, 34). The melatonin dose of 0.5 mg is the optimal dose for eliciting circadian phase shifts (34).

After careful consideration and consultation with colleagues and DoD personnel, treadmill exercise of the proposed intensity and duration was chosen as the stimulus based on findings by our team and others using treadmill exercise. Moreover, treadmills and/or the option of running outside are available on most overseas bases, so running/walking will be generalizable to many scenarios faced by Special Forces, other military, and civilians. We considered “small-space exercise” (e.g., burpees”, push-ups, etc.), which can be the only exercise option for Special Forces in some situations, but decided against it because of lack of evidence for its efficacy for shifting the circadian system.

### Dim red light and placebo comparison treatment

This treatment will involve exposure to dim red light glasses (otherwise identical to the bright light glasses), and a placebo tablet at times corresponding to the timing of bright light and melatonin in the Bright Light + Exercise + Melatonin Treatment. Dim red light has been used as a comparison treatment in multiple bright light studies, and it has no effect on the circadian system. The placebo capsules will also be prepared by the pharmacy and will be identical in appearance and weight to the melatonin capsules.

### End of shift sleep and post-treatment ultrashort sleep-wake schedule

Z machine recorded sleep (8 h) will be repeated on the last night on the shifted schedule. Upon arising, participants will repeat the ultrashort sleep-wake schedule for the final 26 h of the study. For participant safety after expected sleep loss, it will be assured that they have a ride from the laboratory.

### Experimental measures

#### Mental performance: Psychomotor vigilance test

On baseline laboratory Day 1, and on each day of the shifted wake-sleep and light-dark schedule (laboratory Days 4-5), PVT will be measured 5 times across the day (1100, 1400, 1700, 2000, 2300). During the ultrashort sleep-wake schedule (Days 2-3 and 5-6) PVT will be assessed during every wake period (e.g., 0815, 1115, 1415, 1715, 2015, 2315 on Day 2; 0215, 0515, 0815 on Day 3).

In a quiet room with no staff present (to avoid distraction), a 5 min PVT performance test will be performed with a portable monitor (PVT-192, AMI, Inc). The PVT is an electronic test to assess simple reaction time to a visual stimulus. The participant will be given standardized instruction, and will hold the device in a standard way. The participant responds to a visual cue (a display of reaction time in milliseconds) by pressing a button as rapidly as possible upon seeing the cue. Measures will include mean and median reaction time, fastest and slowest 10% responses, and “lapses” (> 500 ms responses).

The PVT is one of the most widely used measures of sleep loss in military (26, 27, 50) and civilian research (51). The PVT is highly sensitive to sleep loss, and PVT shows a profound circadian rhythm (**Figure 2**). The PVT is believed to be highly relevant to many military situations that require maintaining vigilance in conditions of sleep loss and circadian misalignment (50).

#### Pain sensitivity test

During each wake period of the ultrashort sleep-wake schedule, subjects will undergo a test of pain sensitivity. Pressure pain thresholds will be assessed bilaterally over the upper border of the trapezius muscle using a pressure algometer (Wagner FPX 25 Digital Algometer; Wagner Instruments, Greenwich, CT). The trapezius was selected as the primary site to evaluate pain sensitivity because the trapezius is most commonly used site for assessment of pressure pain thresholds, especially in healthy adults (52). Pressure at this site is gradually increased at a steady rate (0.5 kg/sec) and stopped when the subject indicates the stimulus is first perceived as painful. A circadian rhythm in pain sensitivity has recently been established (53). Responses to painful stimuli could have relevancy for many military and civilian scenarios (e.g., injuries, post-surgical pain, chronic pain (54).

#### Physiological performance: Wingate anaerobic test

This test will be performed 4 times (separated by 6 h) during the baseline 26 h ultrashort sleep-wake protocol period (days 2-3), and 4 times during the end-of-study ultrashort sleep-wake protocol (days 5-6). The number of trials is limited to 4 per circadian cycle to reduce the chance of variability associated with fatigue, muscle soreness, or declines in motivation associated with more trials.

For each treatment with n=12, four participants will perform baseline trials 1-4 at 0900, 1500, 2100, and 0300, respectively; 4 participants will perform the trials at 1200, 1800, 0000, and 0600, respectively; and 4 participants will perform the trials at 1500, 2100, 0300, and 0900, respectively. Staggering the trials provides more dispersion of data points across the day (for circadian analysis), and partially distributes fatigue effects across different wake periods. Timing of the Wingate tests will be randomized and stratified by fitness level of the participants (high or low).

We recognize that the trial order for the Wingate Test will not be completely counterbalanced across the time of assessment. Thus, the measured circadian rhythm in performance could be partly masked by fatigue and muscle soreness, as more of latter trials will occur in the morning. In our view, study design features which could avoid this limitation, such as extending the total study duration by 24 h or having participants enter the laboratory at different times, would be more disruptive for addressing the study aims. The literature involving multiple Wingate trials suggest fatigue might not be a significant factor across 4 trials over a 24 h period (55, 56). If there is such an effect, we will be able to quantify it. This factor is also less problematic for our key interest of assessing phase-shifts in the circadian rhythm of Wingate Test performance.

Standard procedures will be followed using a Monark 894E ergometer, with one exception: the Wingate test has traditionally lasted 30 seconds, whereas 6 second tests will be used in the present study. The 6 second test is a validated test to assess peak power output (the primary measure for this test) and results from the 6 second test correlate highly with results from the 30 second test (57).

The seat height will be adjusted according to preference (and the same across trials), and toe clips will be used. Participants will be reminded to give a maximal effort each trial. The participant’s body weight while wearing pre-weighed medical “scrubs” will be measured. Following, a standardized warm-up, 7.5% of the participant’s body weight will be added to the flywheel. The participant will then pedal as fast as possible, pressing a button to drop the 7.5% weight when he/she is ready, which will initiate the 6 second test. Strong verbal encouragement and a time countdown will be provided by research staff. Ergometer software measures include peak power (the highest mechanical power reached during any 3-5 second period) and mean power (average power during the 6 seconds). Participants will then warm-down for 3 minutes.

The Wingate Anaerobic Test is the most widely used test of anaerobic performance in military (58, 59) and civilian research (60, 61). Over the last few decades, it has been increasingly recognized that anaerobic performance is a crucial element of many military situations (62), including sprinting, carrying a heavy load, repetitive lifting, and hand-to-hand combat. A profound diurnal rhythm in Wingate Anaerobic Test performance has been found with peak performance at 1800 h and the worst performance in the morning (60, 61).

##### Sleep: Actigraphy, Z-machine, diary

Disturbed sleep is a hallmark symptom of circadian misalignment (63), and it likely contributes to other consequences of circadian misalignment including sleepiness, and impairments in alertness, cognitive function, and mood impairment. We propose to assess sleep with multiple measures.

#### Actigraphy

Participants will wear wrist actigraphs (AMI, Moitionloggers) continuously (24/7) throughout the two weeks of home recoding and throughout laboratory recording. Actigraphic measures of sleep have been validated against PSG, with minute-to-minute correlations of sleep averaging 85-95% (64, 65). Our team has helped establish this validity, which has been best-established with AMI devices. Acitgraphy is noninvasive and has been an increasingly recognized technology for clinical and research assessment of sleep, with the reliability and sensitivity for detecting improvement in sleep following interventions (66).

Actigraphy has the following advantages for the present study. First, actigraphy will provide an objective measure of home sleep and a screen for normal sleep and a stable sleep schedule prior to entering the laboratory. Second, since wrist actigraphs are worn 24 h per day, actigraphy will provide an objective measure of daytime napping at home and additional verification of absence of napping during the designated wake periods in the laboratory. Third, actigraphy will provide an objective measure of sleep during each of the sleep trials of the ultrashort sleep-wake cycle. Fourth, continuous actigraphic recording allows assessment of nonparametric measures of the rest/activity rhythm, including amplitude and day-to-day stability, which have been associated with circadian synchronization (67), and with health and longevity in some patient populations (68). The following measures will be derived from the all-night actigraphic recording: sleep latency, sleep efficiency, and total sleep time. For sleep measurement during the 1 h sleep intervals of the ultrashort schedule, total sleep time will be quantified. Also calculated will be the amplitude and day-to-day stability of the rest-activity rhythm during the home baseline and the shifted schedule.

#### Z-machine

During each night of home sleep recording and all 4 nights of full-night laboratory sleep recording, sleep will be assessed with the Z-machine (Insight + Model, GeneralSleep.com, Cleveland, OH). The Z-Machine will also record sleep for each of the 1 h sleep periods during the ultrashort sleep-wake schedule on lab days 2-3 and 6-7.

The Z-machine records EEG from only 2 electrodes placed on the mastoid bones (behind the ears) and a ground electrode placed on the center of the neck. The Z-machine has been validated against PSG with sensitivity and specificity of detecting sleep vs. standard technician scoring of 95.5% and 92.5%, respectively (69).

For each night of recording, the Z-machine software will determine total sleep time, sleep latency, and minutes of deep sleep, light sleep, and REM sleep.

#### Sleep diary

Self-reported sleep will be measured with a sleep diary after each night of home assessment and after each night of all-night sleep recording in the laboratory. Participants will answer questions about time of entering bed, time of attempting sleep, sleep latency, number of awakenings and time spent awake for each awakening, total sleep time, time of awakening, time of getting out of bed and sleep quality. Participants will also note on the diary when they remove the actigraph, which will help in interpretation. Self-reported sleep remains the most frequently employed clinical method of assessing sleep quality.

#### Circadian rhythm of urinary 6-sulfatoxymelatonin excretion

To assess baseline and final circadian phase, respectively, urine samples will be collected during the 26 h baseline period and during the final 26 h in the laboratory, while participants are following the ultrashort sleep-wake schedule. Every time a participant urinates during these 26 h periods, a sample will be collected. The time and volume of each sample will be recorded and the sample will be frozen (-70C) for subsequent assays for aMT6s.

#### ELISA assay of urinary 6-sulfatoxymelatonin

The major metabolite of melatonin, urinary aMT6s, will be measured using Novolytix 96 well ELISA kits purchased from ALPCO, Ltd. (Windham, NH). All samples from an individual will be run at the same time on the same plate. In our hands, using this ELISA (previously manufactured by Bühlmann) control urine samples averaging 4–6 ng/ml gave intra- and inter-assay coefficient of variations of 4% and 7%, respectively. From the aMT6s concentration, urine volume, and the collection times, the aMT6s excretion rate (ng/h) will be computed for each collection interval (the interval between one voiding and the next one) and subsequently associated with each 5 min interval within the collection interval. From this time series of 5 min intervals, circadian analyses will be computed.

#### Circadian metrics and shifts

Separate circadian analyses will be conducted for the last 24 h of the baseline ultrashort sleep-wake schedule, and the final 24 h of the end-of study ultrashort sleep wake schedule. Data will not be used for the first 2 h of these schedules to minimize inclusion of acute masking or transient effects that might occur in response to the initial transition to the ultrashort sleep-wake schedule.

Using the least-squares method, the best-fit 24 h cosine function will establish the acrophase (cosine-fitted time of peak), mesor (fitted mean) and amplitude of the circadian rhythms of urinary aMT6s excretion at baseline and end-of study. To further describe changes in phase and waveform of the aMT6s rhythm, we will estimate the circadian timing of the evening rise (onset) and morning decline (offset) of the nocturnal aMT6s peak algebraically from upward and downward crossings of the associated cosine mesor. It is noteworthy aMT6s onset yielded exceptionally clean PRCs to bright light and exercise.

#### Changes in aMT6s phase

Changes in aMT6s phase be calculated by subtracting the end-of-study phase time from the baseline phase time. According to convention, the expected phase delays will be indicated by negative phase shift values. Plasma melatonin and its urinary metabolite, aMT6s, are regarded as the best markers of the central circadian pacemaker of the brain (70). As with our other studies, we have chosen aMT6s because its measurement is relatively noninvasive and does not require medical supervision. From the standpoint of circadian biology, shifts in the circadian rhythm of aMT6s excretion provides the most broadly valuable measure of circadian acclimatization in the proposed study as it most directly reflects the phase timing of the central circadian clock in the brain (suprachiasmatic nucleus of the hypothalamus).

#### Sleepiness and mood

On baseline laboratory Day 1, and all days of the shifted wake-sleep and light-dark schedule (laboratory Days 4-5), sleepiness will be measured with the Stanford Sleepiness Scale (71), and mood will be assessed with the Profile of Mood States (POMS, short form) questionnaire (72) at 5 times across the day. The questionnaire will be administered during each 2 h wake period of the 26 h periods of circadian assessment at baseline and end-of-study (immediately after the 5-min PVT). The composite Total Mood Disturbance Index will be the measure of interest from the POMS. The Stanford Sleepiness Scale is a single-item scale on which participants rate their level of sleepiness “at this very moment” from 1 (“feeling active and vital, alert, and awake”) to 7 (“almost in reverie, sleep onset soon, lost struggle to stay awake”).

The POMS-short form is a 37-item questionnaire in which participants will be prompted to indicate how they feel “right now”. The POMS assesses the intensity of feelings on a 5-point scale (0): not at all; 1 (a little); 2 (moderately); 3 (quite a bit); 4 (extremely). Subscale scores for Tension-Anxiety, Depression-Dejection, Anger-Hostility, Vigor-Activity, Fatigue-Inertia, and Confusion-Bewilderment, and Total Mood Disturbance (TMD) will be calculated.

Sleepiness and impaired mood are hallmark symptoms of circadian misalignment. The Stanford Sleepiness Scale and The POMS questionnaire have been validated against objective assessment of sleepiness (71), and clinical assessment of mood (72), respectively. Moreover, they are commonly used in studies involving repeated measurements, as they are sensitive to experimental interventions. Profound circadian rhythms have been found for these measures, as shown in **Figure 2**.

## Anticipated results

**Figure 5:**
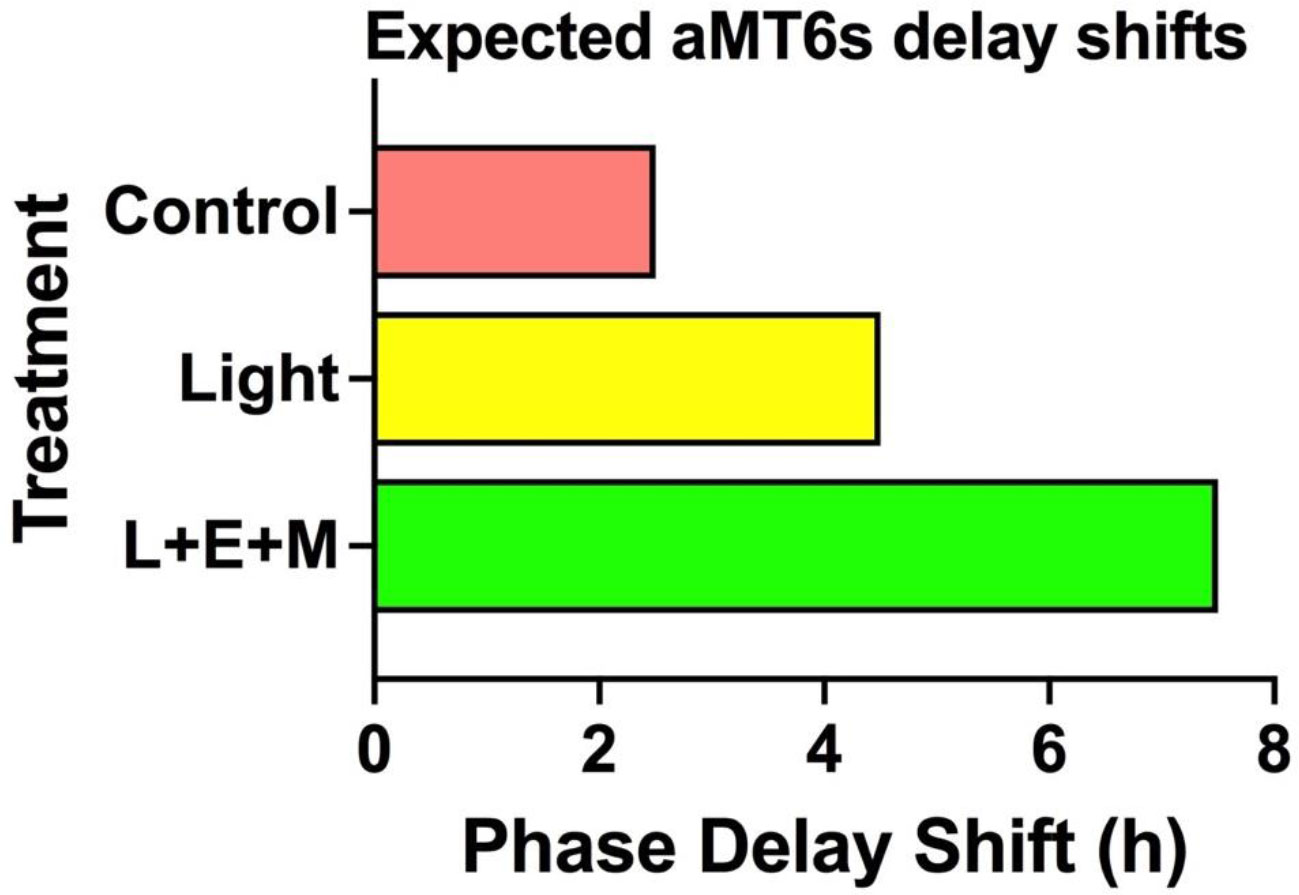
Expected delays in aMT6s phase markers after 3 days of treatment. Compared to corresponding baseline phase we expect delays of 2.5 h the control group, 4.5 h with bright light alone and 7.5 h in the light + exercise + melatonin treatments. Shifts in the our two aMT6s phase markers, onset and acrophase, are expected to be equivalent and significantly correlated.

We expect that compared with the Dim Red Light + Placebo Capsule comparison treatment, circadian acclimation will be significantly greater following Bright Light Alone and Bright Light + Exercise + Melatonin. This will be manifested in greater phase delays in the circadian rhythms in psychomotor vigilance, Wingate Anaerobic Performance, aMT6s, sleepiness, mood, pain sensitivity, and aMT6s, our most direct measure of the acclimatization of the central circadian clock (**Figure 5**). Additionally, we expect the Bright Light + Exercise + Melatonin treatment to elicit significantly less impairment relative to baseline in sleep, psychomotor vigilance, mood, and sleepiness and that compared to the Bright Light Alone treatment the Bright Light + Exercise + Melatonin will elicit significantly greater phase delays in these circadian rhythms (**Figure 5** **),** and result in significantly less impairment in these measures.

### Potential pitfalls and troubleshooting

#### Participant recruitment

Participant recruitment is almost invariably a challenge, particularly for a study that requires a 6.5 day stay in the laboratory. We will utilize multiple sources of recruitment, including social media, flyers, and word of mouth. Moreover, we will recruit from various student outreaches in the Tucson area. A within-subjects design was considered, but it was dismissed as too difficult to get all the participants to perform three 6.5 day laboratory protocols. However, if recruitment lags despite planned efforts some of the participants can be invited to perform multiple treatments, with appropriate statistical consideration and approval from the DoD and IRB. We have performed within-subjects studies previously, and have not found any differences in how participants respond to the protocol in multiple trials. If a participant performs a second treatment, he/she will be randomized to one of the two other treatments; participants will be assigned to a third treatment without randomization. At the study debriefing, we will ask participants if they would be willing to complete additional treatments in the future. Another option, the necessity of which is very remote in our view, would be to forgo testing the placebo control treatment (pending approval), which would still provide important data comparing Bright Light Alone vs. Bright light + Exercise + Melatonin.

#### Participant postponement of laboratory assessment

Unavoidable life events (e.g., illness) can arise. However, frequent postponements of study procedures could harm the execution of the study. The potential for this problem will be reduced by discussing each participant’s schedule early in the recruitment process. Another remedy would be to schedule more heavily during the summer and other breaks from school.

## Discussion

### Statistical analyses

We hypothesize that compared with the Dim Red Light + Placebo Capsule treatment, Bright Light Alone and Bright Light + Exercise + Melatonin will result in significantly greater circadian acclimatization; and that the Bright Light + Exercise + Melatonin treatment will elicit significantly greater circadian acclimatization than Bright Light Alone. Circadian acclimatization will be defined by phase-delays in various circadian rhythms, and by less impairment in sleep and other behavioral functioning on the shifted schedule vs. baseline.

For assessment of circadian rhythms, variables will be assessed during the ultrashort sleep-wake schedule at baseline and at the end of the study. The frequency of assessment will be every time the participant urinates for aMT6s (8-16 times per 24, on average), every 3 h for the psychomotor vigilance test, POMS, and the Stanford Sleepiness Scale, and pain sensitivity; and every 6 h for the Wingate Anaerobic Test.

Less impairment will also be defined by less decline in nighttime sleep during the shifted schedule compared with the baseline night, and less impairment relative to baseline in PVT, Total Mood Disturbance, and Sleepiness. Our primary sleep measure will be sleep duration defined by the Z machine. PVT, Total Mood Disturbance and Sleepiness will be assessed 5 times during the baseline day and 5 times per day during the Days 2-3 of the shifted schedule. The daily mean for each of these variables will be assessed.

### Shifts in circadian phase

A cosine fit of the baseline and end-of study data will be performed using AMI software to determine shifts in the acrophases of the psychomotor vigilance test (median reaction time), the Wingate Anaerobic Test (peak power), the POMS (Total Mood Disturbance), the pain sensitivity test., the Stanford Sleepiness Scale, and the aMT6s excretion rhythm, for which the onset of evening excretion will also be computed.

Planned contrasts will compare phase delays following Dim Red Light + Placebo Capsule vs. Bright Light Alone, and Dim Red Light + Placebo vs. Bright Light + Exercise + Melatonin, as well as Bright Light Alone vs. Bright Light + Exercise + Melatonin. These comparisons will be made by 2X2 treatment by time (beginning vs. end of study) ANOVAs. To control for Type 1 error associated with repeated tests, alpha will be set at 0.0167. Two of the primary measures will be shifts in aMT6s phase and PVT acrophase. Secondary measures will include shifts in the circadian rhythms of Wingate performance, sleep, and mood.

### Impairment on shifted schedule vs. baseline

The primary sleep measure will be total sleep duration on the baseline night and last night of the shifted schedule. First, we will confirm there are no significant between group differences in sleep duration during baseline with a 1-way ANOVA. If there are differences, baseline sleep duration will be entered as a covariate. Effects of treatment on sleep duration will be analyzed by planned contrasts (2X2 ANOVAs) with alpha set at 0.0167. The other variables (median PVT, POMS-total mood disturbance, sleepiness) will be compared with planned contrasts.

Power estimates were made based on n=12 in each treatment, conservative 2-tail tests and a conservative alpha of 0.0167 to adjust for multiple tests. Based on a conservative estimate of phase shift differences of 2 h, and a conservative estimate of standard deviation of 1.5 (we had standard deviations of 1.0 in our PRC studies^7,8^), there is 0.82 power to detect significant difference between treatments.

Based on an estimate of sleep duration declines (baseline to shifted-schedule) varying by 35 min (standard deviation of 24 minutes), we have power of 0.82 to detect significant differences between treatments. Assuming changes in PVT that vary by 25 ms (standard deviation of 19 ms), there is power of 0.81 to detect significant differences between treatments. Likewise, we have power varying between 0.75-0.90 for the other variables.

## Data availability statement

The original contributions presented in the study are included in the article/supplementary material. Further inquiries can be directed to the corresponding author.

## Ethics statement

The study was reviewed and approved by the Institutional Review Board (Human Subjects Protection Program) at the University of Arizona and the Human Research Protection Official of the Department of Defense (United States Special Operations Command). The participants provided their written informed consent to participate in this study.

## Author contributions

The study employing the methods described is in progress and all authors are contributing to its execution. SY and JE conceived the study protocol. SY wrote the first draft of the paper and JE was key in refining and extending the original draft and in the design of the stimulus protocol and methods surrounding collection and analysis of urine samples for quantification the circadian timing of 6-sulfatoxymelatonin. All other authors contributed to the writing or editing of the paper and/or substantially to the testing of participants as we start the study.

## Funding

Study funded by the Department of Defense W81XWH20C0051.

## Conflict of interest

The authors declare that the research was conducted in the absence of any commercial or financial relationships that could be construed as a potential conflict of interest.

## Publisher’s note

All claims expressed in this article are solely those of the authors and do not necessarily represent those of their affiliated organizations, or those of the publisher, the editors and the reviewers. Any product that may be evaluated in this article, or claim that may be made by its manufacturer, is not guaranteed or endorsed by the publisher.
